# Monitoring intracellular metal ion complexation with an acetylene-tagged ligand by Raman spectroscopy†

**DOI:** 10.1039/d0ra06329k

**Published:** 2020-10-01

**Authors:** Seiya Takemura, Hikaru Watanabe, Tatsuya Nishihara, Akimitsu Okamoto, Kazuhito Tanabe

**Affiliations:** Department of Chemistry and Biological Science, College of Science and Engineering, Aoyama Gakuin University 5-10-1 Fuchinobe, Chuo-ku Sagamihara 252-5258 Japan tanabe.kazuhito@chem.aoyama.ac.jp +81-42-759-6493 +81-42-759-6229; Research Center for Advanced Science and Technology, The University of Tokyo 4-6-1 Komaba, Meguro-ku Tokyo 153-8904 Japan

## Abstract

We propose to monitor molecular vibrations to identify metal ion–ligand complexation by means of Raman spectroscopy, which has been applied to track vibrational modes of molecules and to obtain a structural fingerprint. We prepared ligand molecules for Zn^2+^ ion complexation with a dipycolylaminoethyl aniline (DPEA) skeleton and phenylacetylene unit as the Raman tag which showed a typical band around 2200 cm^−1^. Among the labeled ligands synthesized in this study, A-DPEA showed a strong band attributed to the acetylene unit at 2212 cm^−1^, while the addition of Zn^2+^ ion resulted in a band shift to 2220 cm^−1^ due to complex formation. The addition of other metal ions and titration experiments showed that A-DPEA bound with Zn^2+^ selectively with a dissociation constant (*K*_d_) that was estimated to be 0.22 μM. We also conducted cellular experiments and found that complexation between A-DPEA and Zn^2+^ also occurred in cells, with a shift in the Raman signal of the ligand from 2212 to 2215 cm^−1^. Thus, complex formation of the metal ion was identified by monitoring the Raman band shift.

## Abstract 2

Complexation between acetylene-tagged ligand and Zn^2+^ cations was tracked by monitoring changes in molecular vibration.

## Graphical abstract

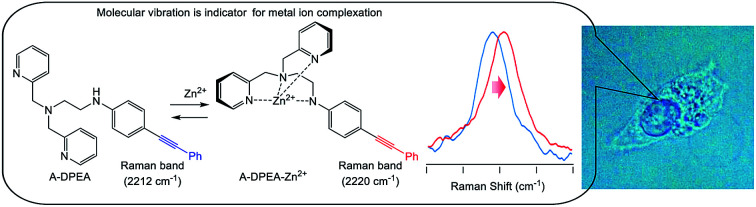

Complex formation with metal cations can determine the function of biomolecules. For instance, proteins with their many reactive side chains have the potential capacity to bind several metal ions and become activated or disactivated.^1,2^ DNA or RNA molecules show enzyme-like properties that can be induced by complexation with metal cations to form second-order structures;^3–5^ indeed, these molecules have been used as building blocks for nanostructures.^6,7^ One of the most useful applications of complex formation is as molecular probes for metal cations in biological systems.^8–10^ A number of ligands bearing reporter units have been designed for sensing of metal cations in living cells or tissue. Complexation is an important and indispensable chemical reaction in living organisms, and therefore, the tracking systems for complex formation have been required to understand their biological roles.

Herein, we make a proposal, in which molecular vibrations are monitored to determine complexation by Raman spectroscopy, which is a standard technique for characterization of vibrational modes of molecules.^11–15^ Recent studies have shown that Raman spectroscopy can provide structural fingerprints of target molecules;^16–18^ thus, we expected that complex formation of metal cations would affect the vibrational modes of its ligand molecules to be determined. Heretofore, a variety of procedures are available to monitor the complexation of metal cations. Representative methods include absorption, fluorescence emission, and NMR spectroscopic techniques to track complexation.^19–23^ Although these spectroscopic methods are useful for characterization, low compatibility of absorption spectra for biological application and the requirement for large-sized substituents for fluorescence emission and a large number of molecules for NMR analysis are issues for these approaches. In this context, we considered that monitoring vibrational modes by Raman spectroscopy may represent a key technology that could be used to identify complexation and to resolve conventional issues because of the advantages that this technique offers, such as the need for small substituents and small volumes (*ca.* 1 μL) to obtain the spectra.

To identify metal ion complexation, we designed ligand molecules for Zn^2+^ ion, the complexation of which could be tracked by monitoring their Raman spectra. We employed a (dipycolylamino)ethyl aniline (DPEA) derivative^24^ as a ligand for Zn^2+^ and modified it with phenylacetylene as a Raman tag because phenylacetylene exhibits a characteristic Raman band even in complex living cell environments.^14,16^ We prepared two modified DPEAs and assessed their complex formation (Fig. 1). Eventually, we could trace the complexation of A-DPEA with Zn^2+^ by monitoring the Raman band shift. In addition, the complexation of A-DPEA in living cells was also successfully monitored.

**Fig. 1:**
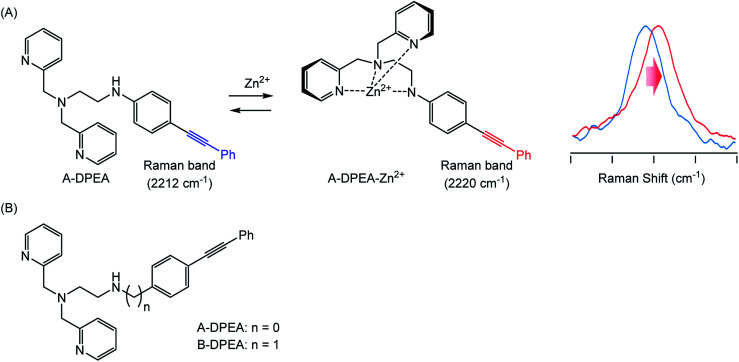
(A) Complexation of A-DPEA and Zn^2+^ ion that results in change of Raman spectra. (B) Ligands bearing Raman tag used in this study.

The synthesis of the ligands bearing a phenylacetylene unit is illustrated in Scheme 1. A-DPEA was synthesized by Sonogashira coupling between phenylacetylene and aromatic bromide 1, which was prepared according to a reported procedure.^24^ We also synthesized a control compound, B-DPEA, in which the phenyl ring and alkylamine were separated by a methylene linkage, by reductive amination of diphenylacetylene 2.

**Scheme 1:**
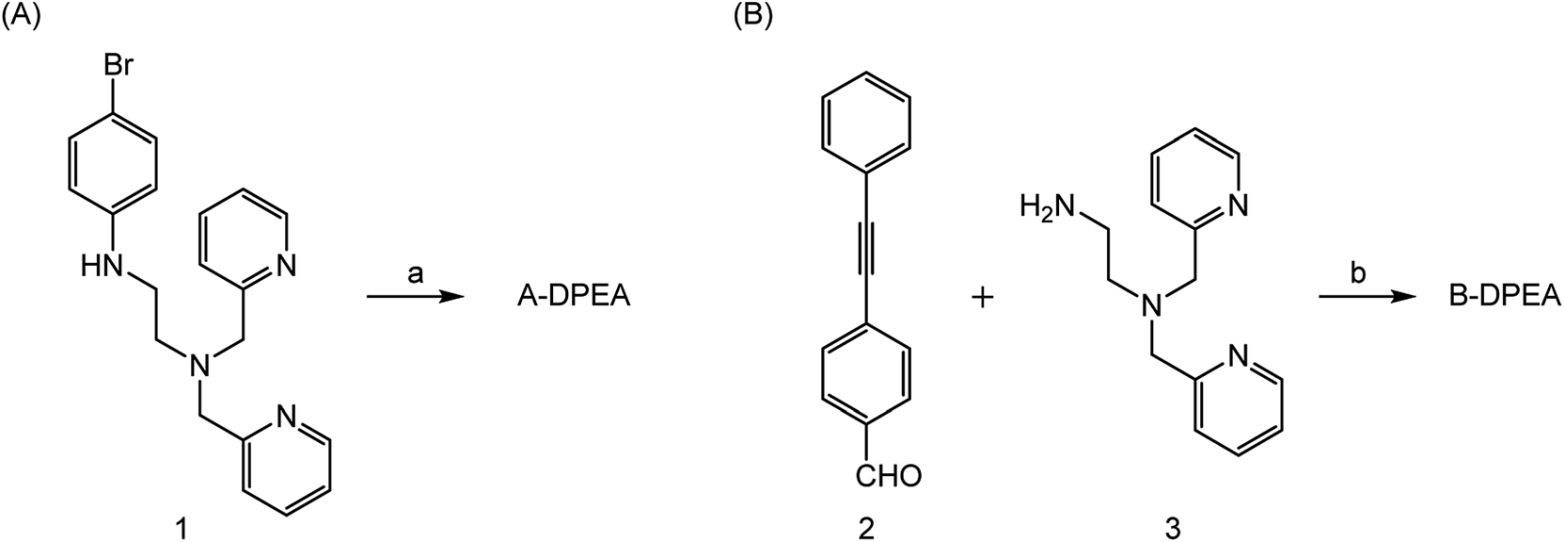
Synthesis of A-DPEA (A) and B-DPEA (B). Reagents and conditions. (a) Phenylacetylene, Pd(PPh_3_)_4_, CuI, Et_3_N, THF, 70 °C, 6%; (b) NaBH_4_, CH_2_Cl_2_–MeOH, 50 °C, 43%.

Initially, we measured the Raman spectra of A-DPEA using excitation at 532 nm. As shown in Fig. 2A, A-DPEA showed a typical band arising from the acetylene group at 2212 cm^−1^ under neutral conditions. As the concentration of Zn^2+^ was increased, the band shifted gradually from 2212 to 2220 cm^−1^ and fully shifted when *ca.* 2 equivalent of Zn^2+^ was added (Fig. 2B). These results indicate that the binding of Zn^2+^ caused a shift of the vibrational frequency of the acetylene unit in A-DPEA. In separate experiments, similar treatment of the control compound, B-DPEA, was conducted, but a negligible change of the Raman band at 2221 cm^−1^ was observed (Fig. 2C). Thus, conjugation between the acetylene unit and binding unit for Zn^2+^ is required for the band shift in the Raman spectra. To assess the complex formation of A-DPEA with Zn^2+^ further, we evaluated the reversibility of the signal change by the addition of a competing agent. Ethylenediaminetetraacetic acid (EDTA), which is a multidentate ligand for Zn^2+^, was added to the complex of A-DPEA and Zn^2+^, and changes of the Raman spectra were monitored (Fig. 3A). The signal of the acetylene unit was shifted from 2220 to 2212 cm^−1^, because the EDTA led to dissociation of Zn^2+^ from A-DPEA. Thus, the binding of Zn^2+^ with A-DPEA is responsible for the band shift in the Raman spectra. We next evaluated the selectivity of A-DPEA toward other metal ions (Fig. 3B). Measurement of Raman spectra revealed that addition of Na^+^, Mg^2+^, Ca^2+^ and Fe^3+^ did not lead to any band shift. Addition of Mn^2+^ and Fe^2+^ resulted in a slight band shift, and addition of Ni^2+^ and Cd^2+^ led to a moderate band shift. Thus, Zn^2+^ was selectively bind with A-DPEA, but several metal ions also showed moderate affinity toward A-DPEA, according to Irving–Williams order.^25^

**Fig. 2:**
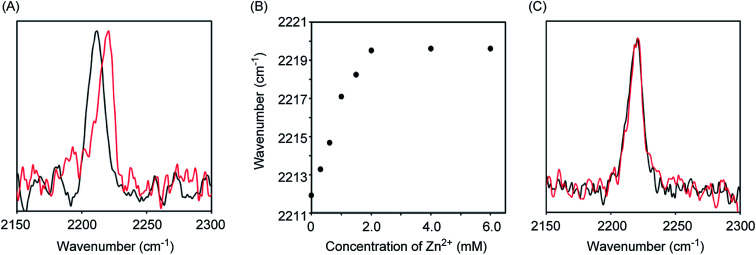
Raman spectra and absorption spectra of A-DTPA and B-DTPA. The Raman spectra were measured using 532 nm excitation. (A) Raman spectra of A-DTPA (1 mM) in the presence (red line) or absence (black line) of ZnCl_2_ (2 mM). (B) Raman signal shift of A-DTPA (1 mM) as increasing Zn^2+^ concentration. (C) Raman spectra of B-DTPA (1 mM) in the presence (red line) or absence (black line) of ZnCl_2_ (2 mM).

**Fig. 3:**
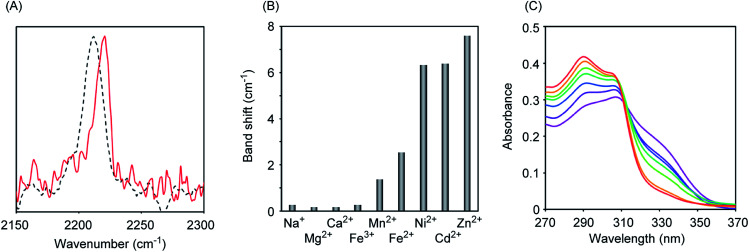
Raman and absorption spectra of A-DTPA. (A) Raman spectra of the complex (1 mM) consisted of A-DPEA and Zn^2+^ in the presence (dashed black line) or absence (red line) of EDTA (2 mM). Ex. 532 nm. (B) Raman band shift of A-DPEA (1 mM) in the presence of NaCl (2 mM), MgCl_2_ (2 mM), CaCl_2_ (2 mM), FeCl_3_ (2 mM), MnCl_2_ (2 mM), NiCl_2_ (2 mM), CdCl_2_ (2 mM) and ZnCl_2_ (2 mM). Ex. 532 nm. (C) Absorption spectra of A-DTPA (10 μM) in the presence of ZnCl_2_ (0 (purple), 2 (dark blue), 4 (blue), 6 (green), 8 (light green), 10 (orange) and 20 μM (red)).

We next measured the absorption spectra of A-DPEA to verify its binding with Zn^2+^. As shown in Fig. 3C, we observed absorption at 310 nm in the absence of Zn^2+^, which is a typical band for the noncomplexed ligand. Addition of Zn^2+^ resulted in a decrease of the absorption at 330 nm, while an increase of that at 290 nm was observed. An isosbestic point was located, which indicates a quantitative change from A-DPEA to the A-DPEA–Zn^2+^ complex. The apparent dissociation constant, *K*_d_ was determined to be 0.22 μM. Thus, A-DPEA retained the binding ability with Zn^2+^, although its affinity for Zn^2+^ was lower than that of the previous modified ligand for Zn^2+^ with the DPEA skeleton,^19^ probably due to the electron-withdrawing properties of the phenylacetylene unit. This low binding affinity of A-DPEA may require the excess amount of Zn^2+^ to complete shift of Raman band in Fig. 2B.

To further assess the function of A-DPEA, we measured the Raman spectra of A-DPEA in the presence of cell lysate. A-DPEA was dissolved in a lysate of a human cell line of fibrosarcoma HT-1080 and lung carcinoma cell line A549. Intense Raman band was observed around 2212 cm^−1^ in both lysates, while addition of Zn^2+^ led to band shift (Fig. S1†). These results are strong indication that complexation of A-DPEA was clearly monitored even in the complicated cellular environments.

For a better understanding of the function of A-DPEA in living cells, we assessed the complexation of Zn^2+^ in a human fibroblast fibrosarcoma cell line, HT1080. The cells were cultured in the presence of A-DPEA for 30 min and, after washing the cells, Raman spectra of the cells were measured. As shown in Fig. 4, the signal arising from the acetylene unit at 2212 cm^−1^ was observed from the cells, while a negligible signal was observed extracellularly. These results strongly indicate that A-DPEA was smoothly taken into the cells and that there are no significant interactions between A-DPEA and cells, because A-DPEA showed the signal at same wavenumber in the extracellular experiments (Fig. 2A). The administration of Zn^2+^ into the cells led to a band shift from 2212 to 2215 cm^−1^. This relatively small band shift (3 cm^−1^) is probably due to a low concentration of Zn^2+^ in the cells, which contributed to the complexation. Thus, the A-DPEA bound with Zn^2+^ in the cells, and it is reasonable to conclude that A-DPEA acts as a reporter molecule for Zn^2+^ complexation even in the cells that can be monitored by Raman spectra.

**Fig. 4:**
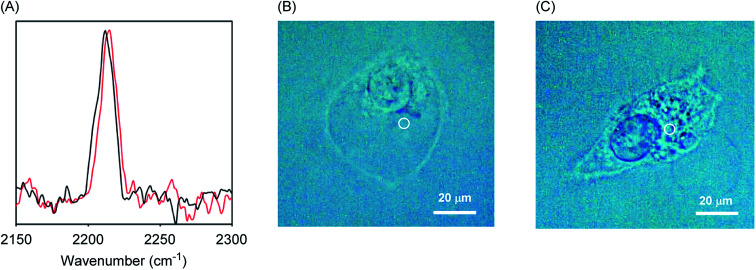
(A) Raman spectra obtained from HT1080 cells in which A-DTPA were taken. The cells were incubated for 30 min in the presence of A-DTPA (100 μM). After wash, ZnCl_2_ (0 mM: black line, 5 mM: red line) was administered to the cells, and then the cells were further incubated for 30 min. After wash, Raman spectra were obtained immediately using 532 nm excitation. (B and C) Images of the cells that were incubated in the presence (B) or absence (C) of ZnCl_2_. White circles indicate the position of irradiation.

## Conclusion

Complexation between ligand and Zn^2+^ cations was tracked by monitoring changes in molecular vibration. The Zn^2+^-binding ligand, A-DPEA, was prepared, to which a phenylacetylene unit was introduced as a reporter tag for the measurement of Raman spectra. A-DPEA showed a band at 2212 cm^−1^ typical of acetylene. Complexation of A-DPEA with Zn^2+^ resulted in a Raman band shift, whereupon the wavenumber was increased by 8 cm^−1^. The addition of EDTA as a competing chelate agent restored the signal to the original wavenumber, and thus the band shift was attributed to the complexation of Zn^2+^. We also applied the present system to monitor the complexation of Zn^2+^ in living cells. A-DPEA was smoothly taken into the cells and the Raman signal of the acetylene unit was easily detected from the cells. The administration of Zn^2+^ to the cells resulted in a band shift due to its complexation in the cells.

In this report, we found that complexation affected molecular vibration of the ligand molecules. This change which attributed to interaction between metal ions and ligand molecules is attractive for the direct observation of complexation, although the low sensitivity and selectivity of the present system should be improved. Resonance Raman spectroscopy or surface enhanced Raman spectroscopy (SERS) may be useful to enhance the sensitivity and intensity of Raman signal, and the improvement of the ligand structure for high performance is in progress.

## Conflicts of interest

There are no conflicts to declare.

## Supplementary Material

RA-010-D0RA06329K-s001
