# Supplementary material for: Monitoring intracellular metal ion complexation with an acetylene-tagged ligand by Raman spectroscopy
Source: RSC Adv. 2020 Oct 1;10(59):36119–23. doi: 10.1039/d0ra06329k (PMC9056985; doi:10.1039/d0ra06329k)
Supplement: RA-010-D0RA06329K-s001 [file RA-010-D0RA06329K-s001.pdf]

Electronic Supplementary Material (ESI) for RSC Advances.
This journal is © The Royal Society of Chemistry 2020

Supporting information

Monitoring intracellular metal ion complexation with acetylene-tagged ligand by Raman spectra

Seiya Takemura,[a] Hikaru Watanabe,[a] Tatsuya Nishihara,[a] Akimitsu Okamoto[b] and Kazuhito Tanabe*[a]

[a]Department of Chemistry and Biological Science, College of Science and Engineering, Aoyama Gakuin University, 5-10-1 Fuchinobe, Chuo-ku, Sagamihara, 252-5258, Japan
[b]Research Center for Advanced Science and Technology, The University of Tokyo, 4-6-1 Komaba, Meguro-ku, Tokyo, 153-8904, Japan

*corresponding author
Phone: +81-42-759-6229 FAX: +81-42-759-6493
e-mail: tanabe.kazuhito@chem.aoyama.ac.jp

Experimental Section

General Methods. Reagents were purchased from Wako pure chemical industries, Tokyo chemical industries, Sigma Aldrich, and nacalai tesque. The course of reactions was monitored by Thin-layer chromatography on silica gel plates (Silica Gel 60 F254). Wakogel 60 was used for silica gel column chromatography. NMR spectra were recorded on a JNM-ECX500Ⅱ (^{1}H : 500 MHz, ^{13}C : 500 MHz) spectrometer (JEOL) and chemical shifts are expressed in ppm downfield from tetramethylsilane, using residual protons in the solvents as internal standard (chloroform: δ 7.26 in ^{1}H NMR, δ 77.0 in ^{13}C NMR Dimethyl sulfoxide: δ 2.49 in ^{1}H NMR, δ 39.5 in ^{13}C NMR). FAB mass spectrometry was performed with a JMS-700A mass spectrometer (JEOL), using nitrobenzyl alcohol as a matrix. Raman spectrum measurement was carried out on a RENISHAW inVia Raman Microscope. UV-visible spectra were obtained at 250-600 nm using a JASCO V-630 UV/VIS spectrophotometer. *N*'-(4-Bromophenyl)-N,N-bis(pyridine-2-ylmethyl)ethane-1,2-diamine (2),[13] and *N',N'*-bis(pyridin-2-ylmethyl)ethane-1,2-diamine (6)[17] were synthesized according to the reported procedures. Human lung carcinoma cell line, HT1080, was purchased from JCRB cell bank (Japanese Collection of Research Bioresources Cell Bank). The cells were incubated using CO_2 incubator (MCO-18AIC, SANYO) at 37 ˚C in 5% CO_2.

Synthesis of A-DPEA. Phenylacetylene (84 mg, 0.82 mmol), tetrakis(triphenylphosphine)palladium (32 mg, 0.027 mmol), and copper iodide (10 mg, 0.050 mmol) were added to a solution of **2** (102 mg, 0.26 mmol) in 4 ml of anhydrous THF and 500 μl triethylamine, and then the resulting mixture was stirred for 3 h at 70 ˚C. After the reaction, the mixture was extracted with chloroform, washed with water then brine, dried over anhydrous magnesium sulfate and concentrated in vacuo. The crude product was purified by silica gel column chromatography (100% chloroform) to give **A-DPEA** (7 mg, 6%) as brown oil. ^{1}H NMR ($CDCl_3$) δ 8.55 (dd, J = 4.0, 1.5 Hz, 2H), 7.60 (ddd, J = 7.5, 7.5, 1.5 Hz, 2H), 7.47 (dd, J = 7.5, 1.5 Hz, 2H), 7.38 (d, J = 8.0 Hz, 2H), 7.32–7.25 (m, 5H), 7.14 (ddd, J = 7.5, 5.2, 1.3 Hz, 2H), 6.50 (d, J = 9 Hz, 2H), 5.15 (brs, 1H), 3.88 (s, 4H), 3.15 (t, J = 5.8 Hz, 2H), 2.87 (t, J = 5.8 Hz, 2H); ^{13}C NMR ($CDCl_3$) δ 149.2, 136.8, 132.9, 132.7, 131.3, 128.3, 127.5, 123.6, 122.5, 122.5, 112.5, 112.4, 110.6, 90.8, 87.1, 60.2, 52.4, 40.9; MS (FAB) m/e 418 [M^+] HRMS (FAB) calcd. for $C_{27}H_{24}N_4$ [M^+], 418.2157 found, 418.2158.

Synthesis of B-DPEA. The mixture of **5** (55 mg, 0.268 mmol) and **6** (65 mg, 0.268 mmol) were dissolved in 2 ml methanol and 2 ml dichloromethane and the resulting mixture was heated to 50 ˚C for 2 h. To the solution of these compounds was added

$NaBH_4$ (30 mg, 0.793 mmol) at 0 ˚C. and then the mixture was stirred for 17 h at room temperature. After the reaction, the resulting mixture was extracted with chloroform, washed with water then brine, dried over anhydrous magnesium sulfate and concentrated under vacuum. The crude product was purified by silica gel column chromatography (chloroform/methanol/NH_3aq = 200/10/1) to give **B-DPEA** (50 mg, 43%) as yellow oil. ^{1}H NMR ($CDCl_3$) δ 8.50 (d, J = 5.0 Hz, 2H), 7.61 (ddd, J = 7.7. 7.7, 1.3 Hz, 2H), 7.52 (dd, J = 7.7, 2.0 Hz, 2H), 7.44 (d, J = 9.1 Hz, 2H), 7.41 (d, J = 9.1 Hz, 2H), 7.35–7.32 (4H), 7.28–7.23 (1H), 7.13 (dd, J = 7.7, 4.5 Hz, 2H), 3.83 (s, 4H), 3.67 (s, 2H), 2.78 (t, J = 6.1 Hz, 2H), 2.74 (t, J = 6.1 Hz, 2H); ^{13}C NMR ($CDCl_3$) δ 159.6, 149.1, 149.0, 136.5, 131.7, 131.6, 128.4, 128.3, 128.2, 123.4, 123.1, 122.1, 122.0, 89.4, 89.3, 60.7, 53.9, 53.3, 46.7; MS (FAB) m/e 418 [M^+] HRMS (FAB) calcd for $C_{29}H_{28}N_4$ [M^+], 432.2314 found, 432.2309.

Measurement of absorption spectra of A-DPEA. A-DPEA 1 **(**10 μM) and $ZnCl_2$ (0-100 μM) in 150 ml of aqueous methanol solutions (methanol : water = 2 : 1, v/v, 100 mM HEPES, pH 7.0) were prepared. Absorption spectra (200-500 nm) were recorded on a JASCO-V630 spectrophotometer at ambient temperature.

Measurement of Raman spectra of A-DPEA and B-DPEA. A-DPEA or B-DPEA (1 mM) and $ZnCl_2$ (0-10 mM) in 2 ml of aqueous methanol solutions (methanol : water = 2 : 1, v/v, 100 mM HEPES, pH 7.0) were prepared. The solution was dropped on a glass slide, then a cover glass was placed on the sample. Raman spectra were measured using 532 nm excitation.

Dissociation of zinc ion from the complex by addition of EDTA. To the complex consisted of A-DPEA-Zn^{2+} **(**1 mM) in aqueous methanol solution (1:1), EDTA (2 mM) was added and then Raman spectra was measured using 532 nm excitation.

Cell culture. Human fibroblasts fibrosarcoma cell, HT1080, was cultured in Roswell Park Memorial Institute media (RPMI1640) supplemented with 10 % FBS, 1 % penicillin-streptomycin, and sodium pyruvate. The cells were maintained at 37 ˚C in 5% CO_2 / 95% air and were kept in a logarithmic growth phase by routine passages every 2−3 days. Prior to the use of cells, the densities of cells were determined using a hemocytometer.

Experiments using cell lysate. A549 cells and HT1080 cells were cultured in 10 dishes (90 % confluent in ϕ100 mm dishes) and washed twice with ice-cold PBS(-). The cell lysate was then harvested by the freeze-thaw process. A-DPEA was dissolved in the cell

lysate containing 1% DMSO and then Raman spectra were measured using 532 nm excitation.

Measurement of Raman spectra in living cells. HT1080 cells (5.0 × 10^3 cells) in the medium of RPMI1640 were seeded onto grass dish. Glass dish was maintained at 37 ˚C in 5% CO_2 / 95% air incubator for 24 h. HT1080 cells were incubated with 0.1 mM A-DPEA in RPMI1640 (DMSO 1%) for 30 min. After incubation, the cells were washed with PBS, and then incubated with 5 mM $ZnCl_2$ in RPMI1640 for 30 min. The cells were washed with PBS, added fresh RPMI1640 (without phenol red) and then Raman spectra were measured by Renishaw inVia Raman Microscope (Renishaw plc, UK). Raman spectra were measured using 532 nm excitation.

Compounds data

A-DPEA(^{1}H NMR)

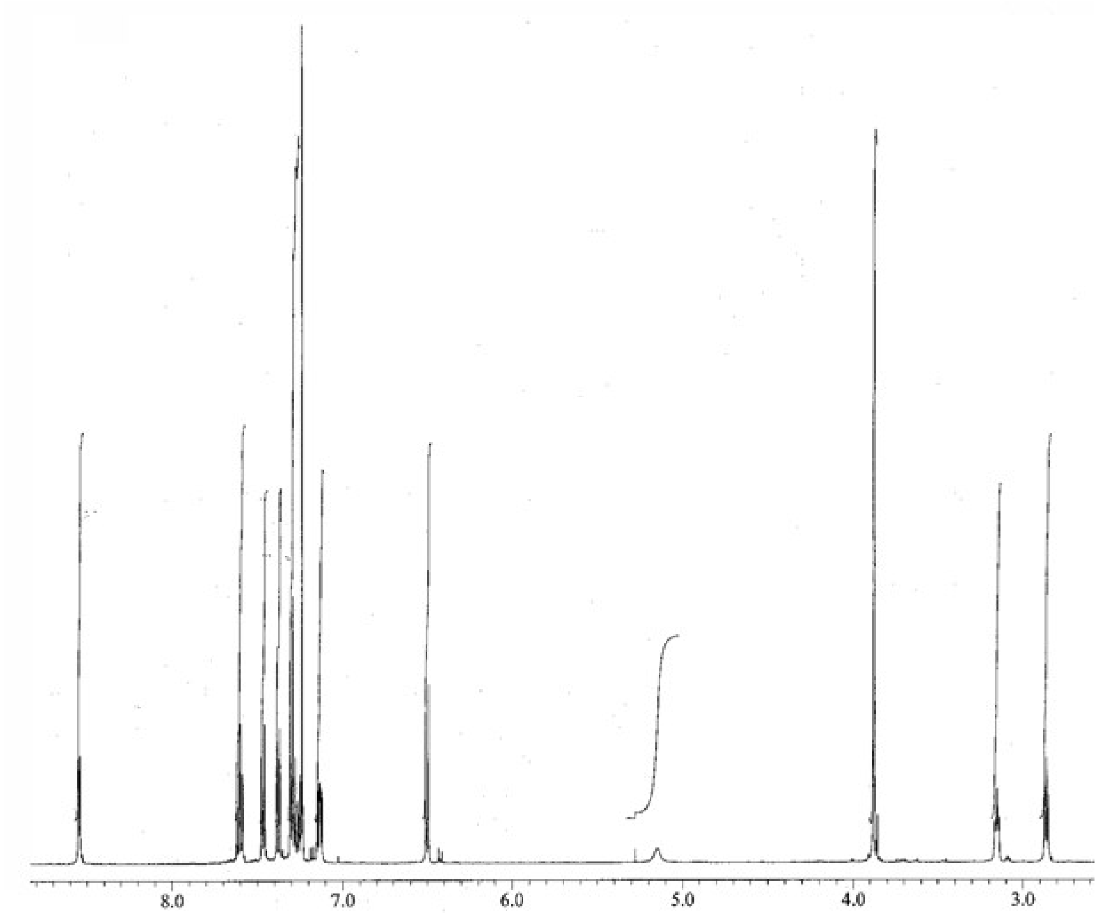

A-DPEA(^{13}C NMR)

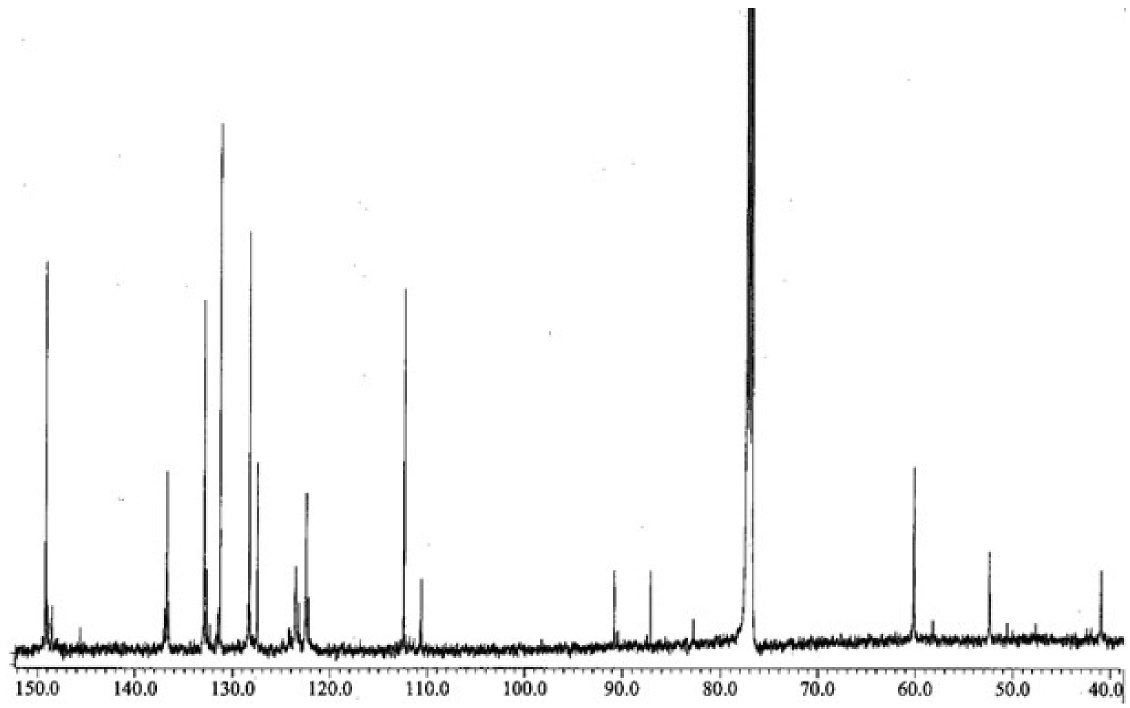

B-DPEA(^{1}H NMR)

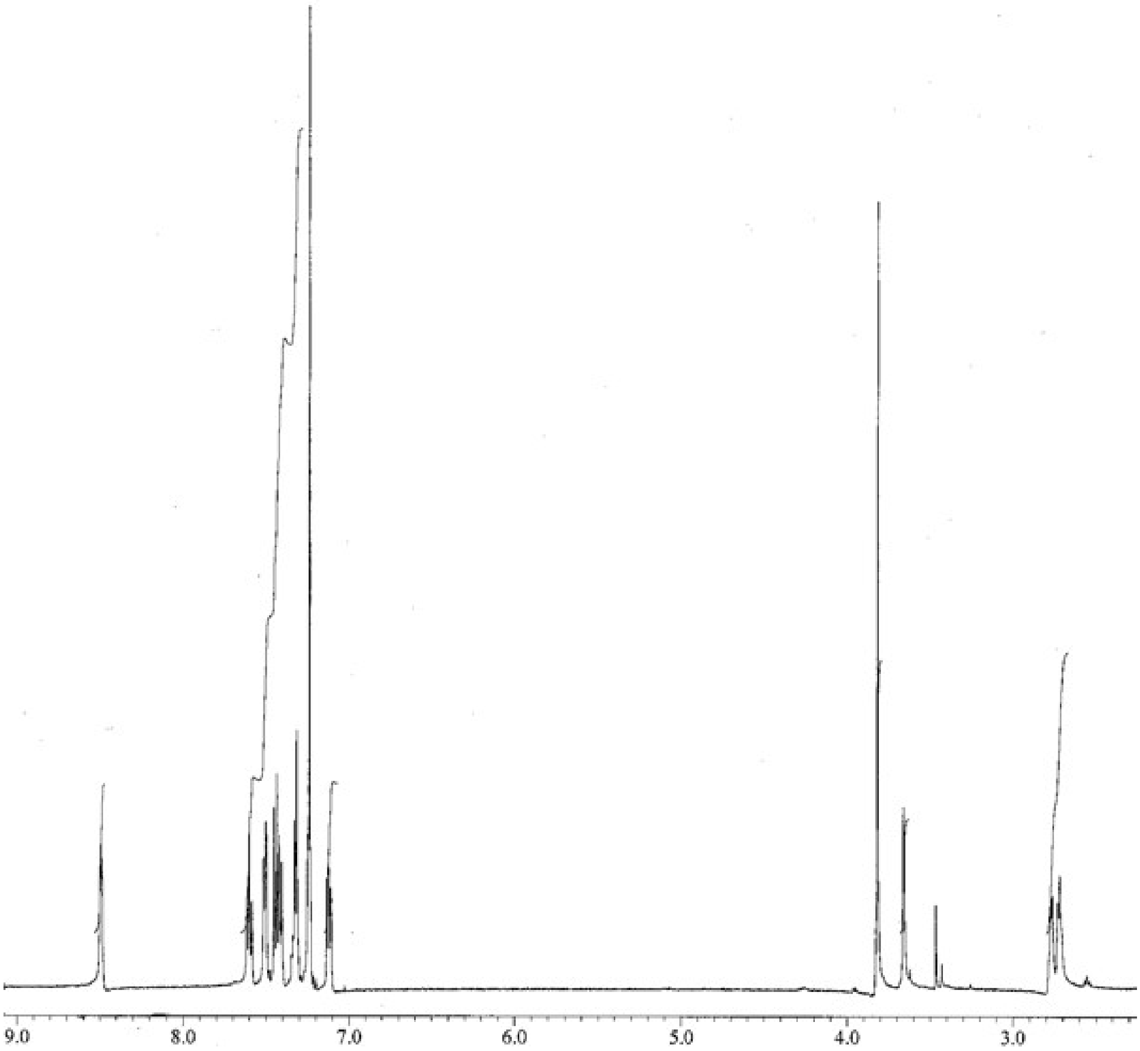

B-DPEA(^{13}C NMR)

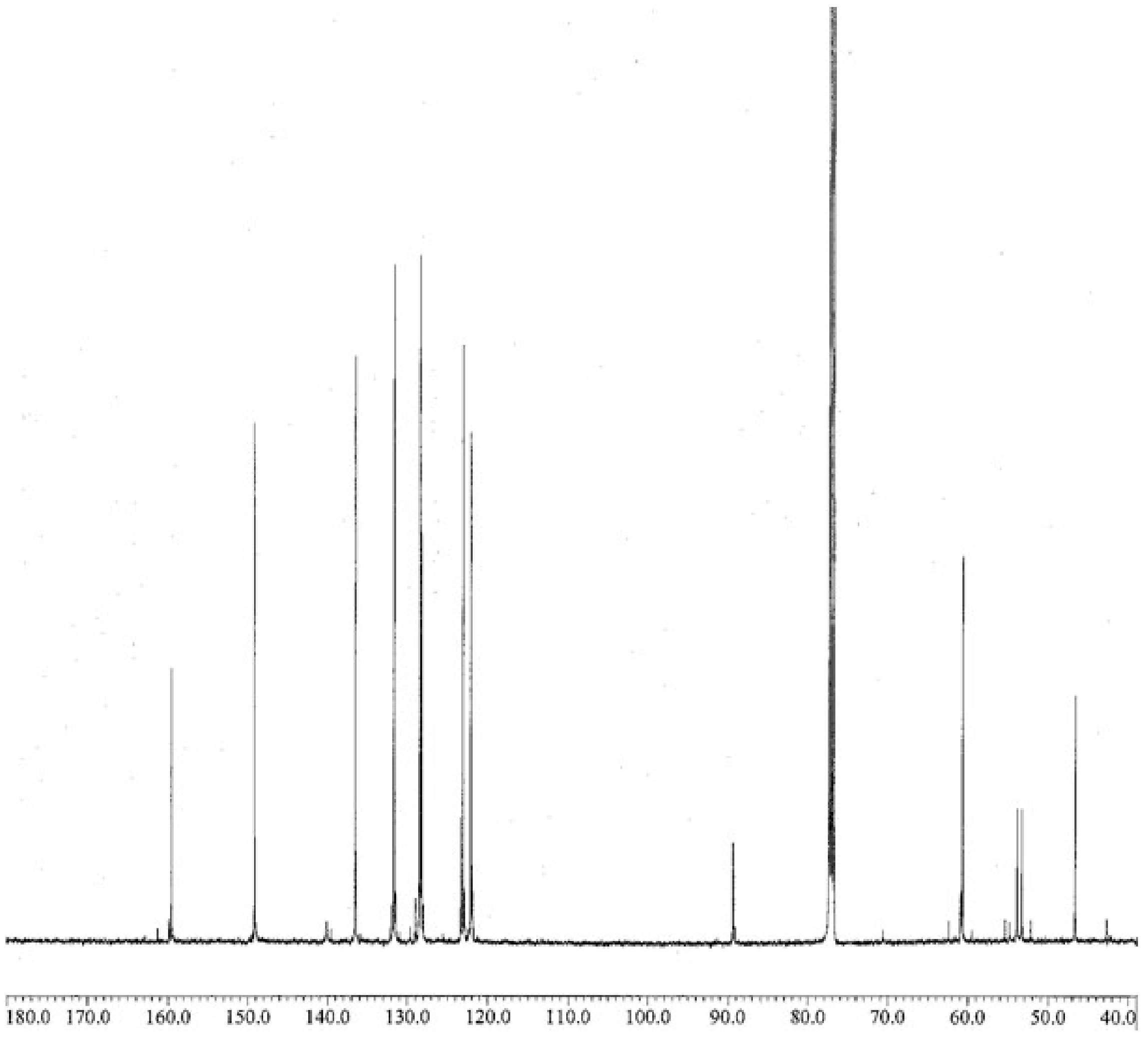

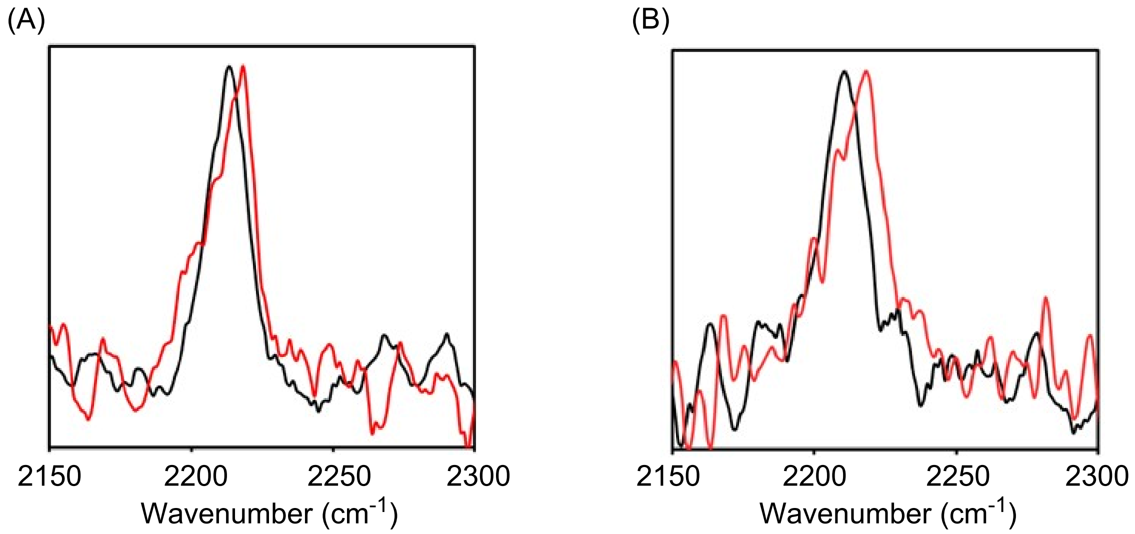

Figure S1. Raman spectra of A-DTPA in cell lysate. Raman spectra of A-DTPA (500 μM) in the presence (red line) or absence (black line) of $ZnCl_2$ (1 mM). The Raman spectra were measured using 532 nm excitation. (A) Lysate obtained from HT1080 cells. (B) Lysate obtained from A549 cells.
